# Supplementary material for: The Impact of Knee Orthoses on Lameness and Weight Distribution in Canine After Rupture of the Cranial Cruciate Ligament
Source: Animals (Basel). 2025 Feb 13;15(4):545. doi: 10.3390/ani15040545 (PMC11852184; doi:10.3390/ani15040545)
Supplement: Supplementary file 1 [file animals-15-00545-s001.zip › animals-3444428-supplementary.pdf]

File S1

1. Assessment, lameness level and measurements of dogs – Questionnaire

Animal Information

Dog's Name: ___________________________________

Breed: ____

Age (years): ____________________________________

Size (top of shoulder blade) (cm): _________________

Body Weight (in kg): _____________________________

Injured limb/joint: _______________________________

Injury: ____

Injury date: _____________________________________

Analgesics/medication:

____

Other conservative treatment methods:

____

Other acute or chronic diseases:

____

Duration and frequency of other conservative treatment methods:

____

Mobility aid:

____

Table S1. Clinical Evaluation.

Criterion	Grade	Clinical Evaluation
Lameness	1	Walks normally
	2	Slightly lame when walking
	3	Moderately lame when walking
	4	Severely lame when walking
	5	Reluctant to rise and will not walk more than five paces
Pain on palpation	1	None
	2	Mild signs; dog turns head in recognition
	3	Moderate signs; dog pulls limb away
	4	Severe signs; dog vocalizes or becomes aggressive
	5	Dog will not allow palpation
Weight-bearing	1	Equal on all limbs standing and walking
	2	Normal standing; favors affected limb when walking
	3	Partial weight-bearing standing and walking
	4	Partial weight-bearing standing; non-weight-bearing walking
	5	Non-weight-bearing standing and walking

Dog's Activity

Average walked distance in a day (km): ______________________

Average number of walks in a day: __________________________

Average duration of one walk (min): _________________________

How does your dog mostly move during walks?

(Slow walk/fast walk/slow run (trot)/fast run (gallop)

How would you rate your dog's current mobility?

(Very good/Good/Moderate/Poor/Very poor)

How would you rate your dog's overall activity level?

(Extremely active/Very active/Moderately active/A little active/Not active)

Researcher's comments:
____

Dog owner's comments:
____

Measurements

1. Measurement Without Mobility Aid

Weight of Front Left Limb: __________

Weight of Front Right Limb: _________

Weight of Rear Left Limb: ___________

Weight of Rear Right Limb: __________

2. Measurement Without Mobility Aid

Weight of Front Left Limb: __________

Weight of Front Right Limb: _________

Weight of Rear Left Limb: ___________

Weight of Rear Right Limb: __________

3. Measurement Without Mobility Aid

Weight of Front Left Limb: _________

Weight of Front Right Limb: ________

Weight of Rear Left Limb: __________

Weight of Rear Right Limb: _________

1. Measurement With Mobility Aid

Weight of Front Left Limb: _________

Weight of Front Right Limb: ________

Weight of Rear Left Limb: __________

Weight of Rear Right Limb: _________

2. Measurement With Mobility Aid

Weight of Front Left Limb: _________

Weight of Front Right Limb: ________

Weight of Rear Left Limb: __________

Weight of Rear Right Limb: _________

3. Measurement With Mobility Aid

Weight of Front Left Limb: ________

Weight of Front Right Limb: _______

Weight of Rear Left Limb: _________

Weight of Rear Right Limb: ________
